# Applying intervention mapping to develop a program for promoting short physical activity breaks during class time in upper secondary schools: the MOVE12 protocol study

**DOI:** 10.3389/fspor.2024.1460373

**Published:** 2025-01-13

**Authors:** Svein Barene, Patrick Foss Johansen, Hege Eikeland Tjomsland, Rolf Inge Ølberg, Miranda Thurston

**Affiliations:** ^1^ Department of Public Health and Sport Sciences, University of Inland Norway, Elverum, Norway; ^2^ Department of Sport, Food and Natural Sciences, Western Norway University of Applied Sciences, Bergen, Norway; ^3^ Department of Public Health, Østfold County Council, Sarpsborg, Norway

**Keywords:** school-based physical activity, intervention mapping, health promotion, high school, adolescents

## Abstract

**Introduction:**

Physical inactivity is a global health challenge, exacerbated by increased screen time and sedentary behaviors. Enhancing physical activity levels at schools offers a promising approach to promote lifelong healthy habits.

**Methods:**

This protocol paper outlines the MOVE12 pilot study, a 12-week intervention study designed to increase physical activity among Norwegian upper secondary school students through 6–7-min daily MOVE-breaks integrated into lessons. Developed using the six-phase Intervention Mapping (IM) protocol, grounded in the social-ecological model and self-determination theory, the intervention focuses on fostering motivation and creating a supportive environment. Key steps include needs assessment, performance objectives, theoretical methods, and program structuring for sustainability.

**Results:**

Linear mixed models, *t*-tests, and regression analyses will evaluate quantitative outcomes, while qualitative focus groups will explore engagement and behavior change.

**Discussion:**

MOVE12 provides insights into scalable school-based interventions addressing physical inactivity, highlighting the potential of the IM framework to establish sustainable health promotion strategies.

## 1 Introduction

Physical inactivity is associated with numerous non-communicable diseases and substantial economic costs globally (1, 2). Regular physical activity among children and young people has been associated with physical fitness, cardiometabolic health, bone health, cognitive outcomes such as academic performance and cognitive function (3, 4), as well as mental health (5–7), quality of life (8), and, to some extent, mental well-being (9).

Given such evidence, the World Health Organization (WHO) (10) has advocated for increasing physical activity and reducing sedentary behavior, particularly among young people, as part of preventive measures against non-communicable diseases. However, global physical activity levels remain below the WHO's recommended minimum of 60 min of moderate-to-vigorous physical activity per day for children and adolescents, with significant declines throughout childhood and adolescence, especially among lower socioeconomic groups, a trend that often persists into adulthood (11). The rise of digital platforms and increased screen time in recent years has exacerbated concerns about sedentary behaviors among children and adolescents (12). Although trends are complex, this shift towards inactivity in future generations could have detrimental impacts on their physical and mental health (13). Furthermore, sedentary behaviors may have persisted at elevated levels following the COVID-19 pandemic compared to before (14). The UN's 2030 Agenda for Sustainable Development also underscores youth health as a priority focus area, highlighting the urgency of addressing this issue.

In Norway, health authorities recommend that children and adolescents engage in at least 60 min of daily physical activity at moderate to high intensity, while also reducing sedentary behavior (15). Schools have been identified as critical venues for promoting regular physical activity, serving as the only mandatory setting for such activities until the age of 19 in many countries. However, the scope of compulsory physical education is limited. In Norway, for instance, this equates to just one session per week, with an effective duration of approximately 70–80 min. The typical timetable in Norwegian secondary schools consists of 90-min instructional blocks interspersed with 10–15-min breaks devoid of structured physical activity. During these intervals, most students remain inactive, often engaged with social media on their mobile devices (16).

In 2021, the Norwegian government introduced a policy for the gradual inclusion of daily physical activity within school programs, providing schools with the discretion to tailor these activities independently (17). In support of this policy, leading health organizations joined forces to create a political platform named the “Alliance for Physical Activity in Schools,” encapsulated by the motto: “Daily physical activity in schools for all students” (18). Although some review articles suggest that physical education classes, after-school sports programs, and accessible sports facilities in schools are linked to higher levels of physical activity (19), the observed effects are generally modest, and it remains unclear whether these improvements are sustained over time (11, 20).

Recent empirical evidence further underscores both the potential and complexity of school-based interventions. For example, the “Join the Healthy Boat” program in Germany aimed to reduce children's sedentary behavior but did not achieve significant reductions despite decreasing screen time (21). In contrast, a combined physical activity and nutrition intervention in the Netherlands led to improvements in children's moderate-to-vigorous physical activity (MVPA) levels and BMI (22). Similarly, school-based interventions in disadvantaged neighborhoods in France demonstrated positive effects on children's physical activity by targeting multiple levels of the socioecological model (23).

Previous studies have identified several problems relating to the implementation of school-based programs (24, 25), which are often conceptualized as common barriers to promoting physical activity in schools (26). These can be roughly divided into institutional factors (such as school policies, facilities, and administrative support), teacher-related factors (including teachers' beliefs and skills), and student-related factors (such as characteristics of the student population). These barriers are often specific to the school level (primary vs. upper secondary) and the level of teacher experience (specialist vs. non-specialist) (27). Moreover, a systematic review by Cassar et al. on the implementation of school-based physical activity interventions found that implementation models are often used primarily for interpreting results and analyses rather than being employed as planning tools throughout all study phases. This limited application of implementation models may contribute to the modest success of interventions in real-world conditions (24). Additionally, there is an increasing awareness of the importance of understanding implementation in terms of sustainability (28) and equity (20).

Given the widespread decline in adolescent physical activity and the need for systematically planned, evidence-based interventions, this study aimed to thoroughly document the planning process of a 12-week physical activity pilot intervention (MOVE12) using the Intervention Mapping (IM) protocol. The IM protocol emphasizes understanding the determinants of behavior and environmental conditions (29), and is guided by the social-ecological model (SEM). Detailed descriptions of such protocols are often missing in study designs, yet they are crucial for mapping intervention development, addressing identified problems, and testing potential solutions (29). The primary objective of MOVE12 is to promote short physical activity sessions (MOVE-breaks) during class time in Norwegian upper secondary schools (16–17 years of age), potentially leading to sustained health benefits and encouraging a more physically active lifestyle outside of school.

## 2 Materials and methods

For the systematic development of the MOVE12, the stepwise methodology of the IM protocol was employed. IM is a widely recognized methodology for planning health promotion programs that are both theory-based and evidence-based (30). The IM protocol comprises six steps: (1) conducting a needs assessment, (2) defining performance objectives and creating a matrix of change objectives, (3) selecting theory-based intervention methods and practical applications, (4) organizing these methods and applications into an intervention program, (5) planning for the adoption, implementation, and sustainability of the program, and (6) generating an evaluation plan (30). It is important to note that the application of the IM protocol in the MOVE12 was not strictly linear as the six steps suggest. Instead, the process was characterized by iterative refinement through ongoing discussions and interactions with the literature and the planning group. This iterative approach involved moving back and forth between different steps, continuously revising and refining the methodology as new information and insights emerged. This flexibility is a fundamental aspect of the IM protocol, as emphasized by Eldredge et al., who state that program developers often “move back and forth between tasks and steps as they gain information and perspective from various activities” (29). Consequently, while the IM framework provides a structured guideline, its application in practice requires adaptation and responsiveness to the evolving context and feedback received during the planning and implementation phases.

The initial step in the IM process involved establishing a planning group that included key stakeholders to ensure comprehensive collaboration across all levels of the initiative. The MOVE12 planning group included diverse representatives: two from the current county municipality (the Head of the Department of Public Health and the Project Manager for MOVE12, three from the research team [the principal investigator (PI) from Inland Norway University of Applied Sciences (INN) and two from Østfold University College], and two from each participating school (a leader and a teacher ambassador with designated resources to support the implementation process). The role of the teacher ambassadors was to serve as key mediators between the project team and the schools throughout all three phases. They were previously engaged by the county municipality as part of a broader school health initiative focusing on sleep and nutrition that began in 2017. This prior engagement was both convenient and advantageous, as it enabled collaborative relationships to be formed with the participating schools, helping to foster a sense of ownership and commitment to the project at each individual school.

### 2.1 Logic model of the problem: why are young people in upper secondary school inactive?

The initial step of the IM protocol involved developing a logic model to graphically represent causal relationships and guide the planning team in addressing key health challenges (30). This process was informed by the public health profile of the current county municipality and a comprehensive needs assessment, integrating evidence from multiple data sources. For example, the 2022 Youth Profile (31) for one of the counties contributing half of the study's participants revealed a higher proportion of adolescents living in households with persistent low income (<60% of median household income) compared to the national average. This socio-economic disadvantage significantly impacts life and developmental opportunities, resulting in lower performance on cognitive and language tests, reduced academic motivation, and diminished self-efficacy, increasing the risk of school dropout. Additionally, these adolescents face a heightened risk of future physical and mental health challenges, underscoring the need for targeted interventions to improve well-being and educational outcomes.

A targeted literature review of peer-reviewed studies and reports on adolescent physical activity behaviors identified key barriers, including insufficient knowledge, low self-efficacy, and inadequate school support. Regional public health data highlighted local trends in adolescent physical inactivity, emphasizing the urgency of addressing sedentary behaviors during school hours.

Stakeholder engagement with school leaders, teacher ambassadors, and public health representatives informed the intervention's practical components, addressing curricular constraints and equipment needs. The demographic diversity of students across schools and academic tracks (academic and vocational) ensured the intervention was tailored to varied educational contexts and social dynamics. This multi-faceted approach ensured a robust, evidence-based, and context-sensitive intervention design.

**Figure 1:**
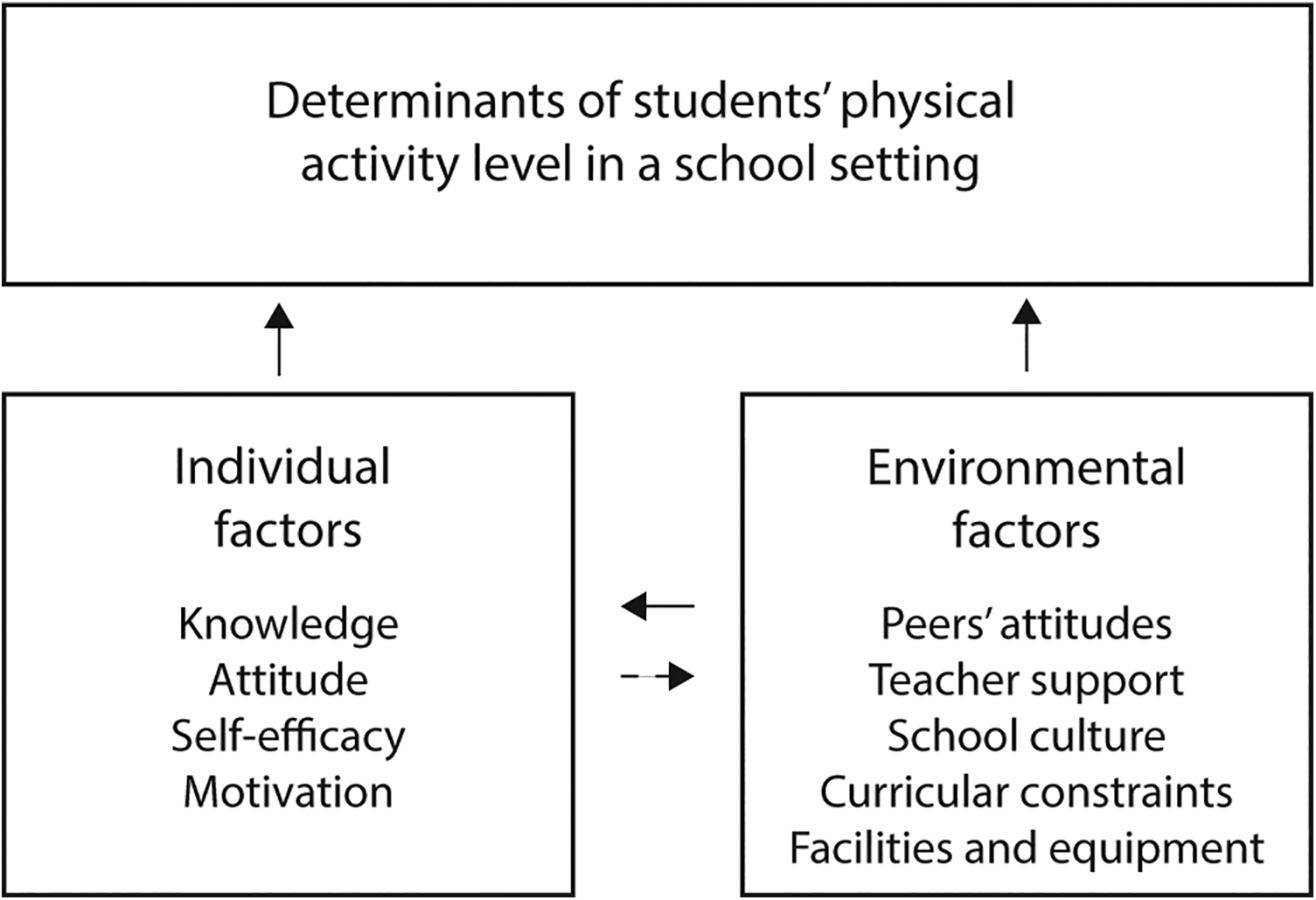
Logic model of the problem. Factors influencing students’ physical activity level at school.

According to Figure 1, adolescents' engagement in physical activity at school is significantly influenced by intrapersonal factors such as their knowledge, predispositions, and previous experiences with physical activity (32). Key to this is providing students with up-to-date knowledge about the importance of physical activity for psychosocial well-being and maintaining good health. This educational aspect, combined with the unique format of MOVE12 (student-led sessions in pairs), aims to foster positive attitudes towards participation (33). Additionally, self-concept plays an important role in shaping how they perceive their abilities and the value they place on physical activity (34, 35). The MOVE12 includes exercises that all students can master regardless of their initial skill level, enhancing a sense of self-efficacy among students who may traditionally be reluctant to engage in physical activities (36, 37). Furthermore, for adherence to the program, it is vital that students find the activities enjoyable (motivation), which is facilitated by allowing some freedom in choosing the activities they participate in. According to self-determination theory (SDT), fulfilling the basic psychological needs of competence (feeling effective and mastering challenging tasks), autonomy (having a sense of initiative when participating voluntarily), and relatedness (feeling accepted and integrated within a social context) enhances adolescents' well-being and intrinsic motivation (38).

At the interpersonal and organizational levels, several key factors have been identified as influencing physical activity levels among school-aged youth. Research highlights that social influences, including peer attitudes, can negatively impact not only adolescents’ physical activity levels but also their self-concept (39, 40). Moreover, the lack of adequate support from teachers and school leaders can further inhibit active engagement (35, 39, 41). These elements are essential in establishing a positive culture around class participation rates and individual student engagement, a responsibility that largely rests with the school management. To successfully implement MOVE12, it is crucial that school management collaborates with teachers to find effective ways to accommodate short MOVE-breaks during class time within a busy school schedule characterized by prolonged sedentary periods (curricular constraints) (42). Lastly, it is essential to address challenges such as limited access to appropriate equipment and suboptimal facilities, as these constraints could hinder the implementation of MOVE-breaks (37, 39).

### 2.2 Logic model of change

**Figure 2:**
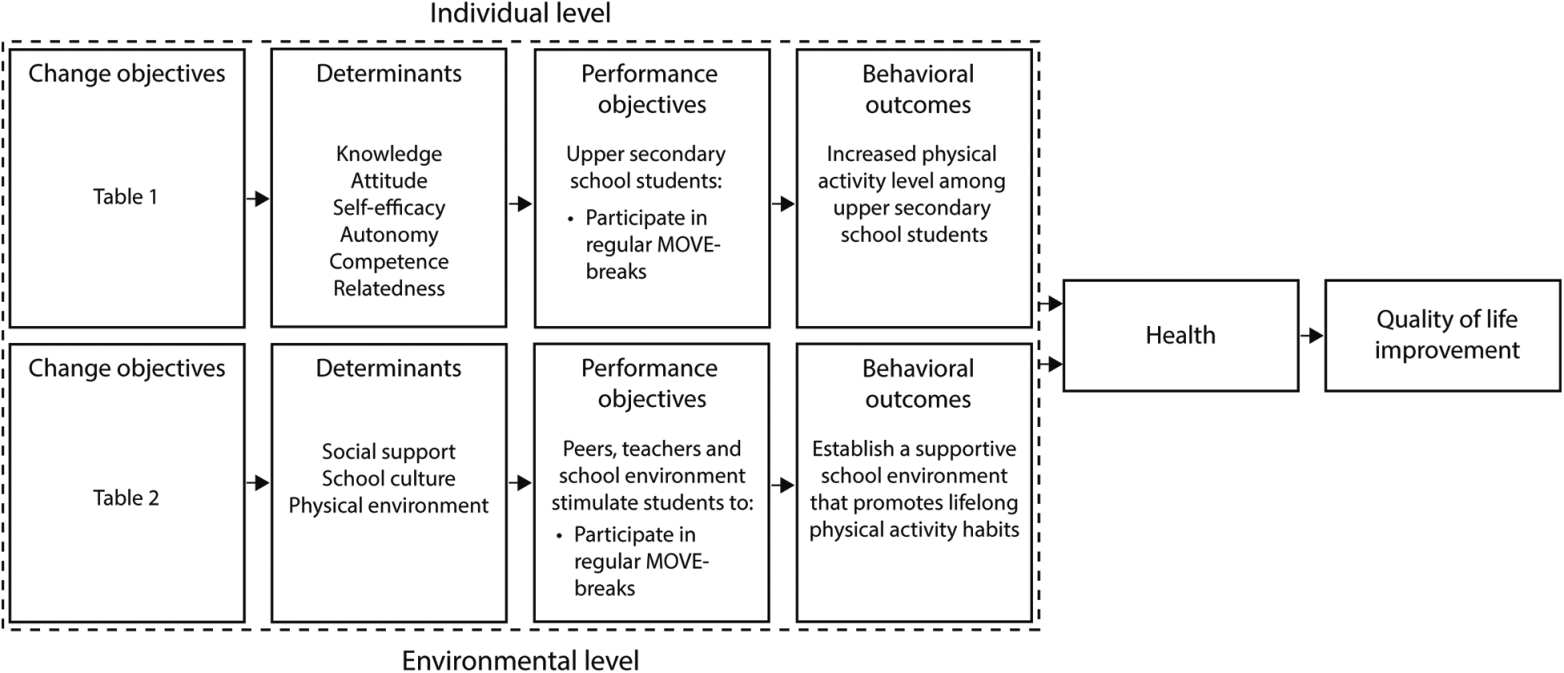
Logic model of change of the intervention.

The second step of the IM protocol process focused on identifying the targeted changes at both behavioral (who will change) and environmental (what will change) levels based in relation to the understanding of the problem outlined in Step 1. This was achieved by creating a logic model of change (Figure 2) that integrated elements from the social-ecological model (SEM) and self-determination theory (SDT). The SEM serves as a comprehensive framework that can be applied to help elucidate the complex interplay between individual and environmental factors in shaping behaviors at micro, meso and macro levels. Specifically, within the realm of physical activity, SEM underscores that individual behaviors are influenced not only by personal decisions but also by environmental factors such as social support, the physical environment, institutional policies, and community norms (43). Meanwhile, SDT focuses on the assumed psychological needs for autonomy, competence, and relatedness. By addressing these needs through physical activity in the school organizational context, SDT provides a foundation for designing interventions that enhance motivational factors, making physical activity both personally appealing and supported by social contexts (38).

At the individual (micro) level, the intervention focuses on enhancing intrinsic motivators for students' physical activity (PA) throughout the school day by promoting enjoyable and engaging activities. This strategy aims to cultivate lasting PA habits, which can improve health and overall quality of life. At the environmental (meso-organizational) level, the goal is to establish a supportive school environment that encourages lifelong physical activity habits. This involves integrating effective, inclusive, and multicomponent school-based interventions that enhance access to safe and appealing PA settings (36, 41). At the macro level, the intervention seeks to engage stakeholders, including local authorities, educational institutions, community organizations, and policymakers, to create supportive environments for physical activity in schools. It advocates for increased funding, improved infrastructure for accessible and safe exercise facilities near classrooms, and the integration of school-based physical activity into public health and education policies. Additionally, the project aims to raise awareness of schools as vital arenas for health-promoting physical activity through local, regional, and national initiatives. By fostering cross-sector collaboration, the project aims to achieve sustainable, systemic changes that encourage active lifestyles and enhance youth well-being.

In accordance with the IM protocol methodology, the final outcomes of the Move12 were subdivided into various components, with the desired changes in these components articulated as performance objectives (Figure 2). Based on the needs assessment derived from our literature review, the primary performance objective was to encourage students to participate in 1–2 daily MOVE-breaks, physical activity sessions lasting 6–7 min, integrated into 90-min class periods during regular school days. Several determinants identified through the IM process, were recognized as critical to achieving this objective. At the individual level, changeable determinants of PA participation, as identified in our literature review, included knowledge, attitude, and self-efficacy, as well as the three basic psychological needs: autonomy, competence, and relatedness. Stakeholder feedback, particularly from teacher ambassadors, revealed that while the initial objective focused on student participation and recognizing the benefits of MOVE-breaks, greater autonomy in activity planning was necessary to increase motivation. This led to a revision in which students were allowed to select activity content within structured guidelines, exemplifying how the IM protocol steps were iteratively refined. At the environmental level, modifiable determinants included social support, school culture, and the physical environment. Feedback from stakeholders highlighted logistical constraints in classroom environments. To address this, we refined the objectives to incorporate practical facilitation measures, such as introducing simple equipment like beanbags and dice, to expand the range of accessible and engaging activities. Following the IM protocol methodology, matrices of change objectives were developed by intersecting performance objectives with these determinants (Table 1). These matrices outlined the necessary achievements for meeting each performance objective. For example, one key change objective at the individual level was ensuring that students understood the potential benefits of participating in MOVE-breaks (knowledge). Feedback during the planning phase reinforced the importance of aligning these objectives with both student needs and environmental realities, demonstrating the iterative nature of the IM protocol.

**Table 1:** Performance objectives and determinants for MOVE-break participation.

Performance objectives		Determinants					
		Knowledge	Attitude	Self-efficacy	Autonomy	Competence	Relatedness
Individual level	Students participate in 1–2 daily MOVE-breaks.	Students know potential effects of participating in MOVE-breaks.	Students have a positive attitude towards participating in MOVE-breaks.	Students are convinced they can overcome barriers to participate in MOVE-breaks.	Within certain limits, students can choose groups and activity content themselves.	Are aware of the purpose and competence targets.	Receive social support from their peers, the PE teacher and school’s teacher ambassador
		Social support		School culture		Physical environment	
Environmental level	Peers, teachers and environment stimulate students to participate in MOVE-breaks.	Peers and teachers motivate students to participate in MOVE-breaks.		Peers and teachers and school leaders have a positive attitude towards MOVE-breaks.		Schools are actively creating and supporting opportunities for MOVE-breaks during class time.	

### 2.3 Theory-based intervention methods and practical applications

The third step of the IM process focused on selecting theory-based intervention methods and translating them into practical applications tailored to the MOVE12 intervention (Table 2). These applications were informed by insights gained from the comprehensive needs assessment and the Public Health Profile (31) described in Step 1. For instance, the 2022 Youth Profile highlighted persistent low household income and its adverse effects on adolescents' cognitive performance, motivation, and self-efficacy, which informed specific methods and applications. For example, goal-setting activities were introduced to foster autonomy by encouraging students to collaboratively plan MOVE-break sessions, selecting activities that aligned with their personal interest. Guided practice with feedback was used to build self-efficacy, with PE teachers and teacher ambassadors providing regular feedback during preparatory practice sessions. To enhance relatedness, social support was mobilized through peer encouragement, and teacher ambassadors served as key contacts to support students throughout the intervention. These methods were iteratively refined to align with the intervention's goal of increasing physical activity among diverse student populations.

**Table 2:** Theory-based methods and applications for enhancing MOVE-break participation.

Measure	Determinants	Theory-based methods	Practical application
Individual level	Knowledge	Information meetings (44) Advance organizers (52)	The purpose and potential benefits of the MOVE-breaks are communicated by the Project leader and the PI to the students. Presenting an overview of the different exercises in a digital compendium.
	Attitude	Direct experience (45)	Encouraging a process whereby knowledge is created, and attitude is changed towards participating in MOVE-breaks through the interpretation of experience.
	Self-efficacy	Guided practice/Feedback (46, 47)	PE-teacher will provide feedback to the students related to the 2 weeks practice prior of the start of the intervention. The teacher ambassador will provide regular feedback to the students related to MOVE-breaks they are responsible for organising.
	Autonomy	Goal setting (48)	Students pair plan the content of the MOVE-breaks sessions, including a definition of which health-related characteristics and skills they want the session to help influence.
	Competence	Guided practice (46)	The students, both in their role as instructors and as participants in others’ sessions, will have sufficient time for demonstration, exercise and rehearsal with feedback from the PE teacher and teacher ambassador.
	Relatedness	Social support (49)	Both peer students, the sports teacher and the school's teacher ambassador will provide support throughout the intervention period.
Environmental level	Social support	Mobilizing social support (49)	Each participating school will be provided with financial incentives to appoint a dedicated teacher ambassador. This ambassador will act as the key contact between the research team and the school, specifically tasked with facilitating the MOVE-breaks and supporting students throughout the project. Additionally, both school management and teachers will be encouraged to support and make it possible for the students to be physically active through MOVE-breaks in selected teaching sessions.
	School culture	Tailoring (44) Sense-making (50) Financial incentive	The school management defines MOVE-breaks as a special focus area which is communicated through information letters to parents and guardians. The school, through its management, teacher ambassadors and teachers promote and arranges for MOVE breaks to become a natural part of the school's daily operations. Enable the school to engage a dedicated teacher ambassador with responsibility for following up students and teachers at each school.
	Physical environment	Facilitation (51)	The classroom environment will be facilitated with adequate equipment that makes physical activity possible.

To strengthen students' self-efficacy, guided practice sessions with feedback from teacher ambassadors were incorporated, addressing the identified need for supportive school structures. Pairing students to co-lead MOVE-breaks fostered autonomy and relatedness, targeting the psychological needs for competence, autonomy, and social connectedness highlighted in Step 1.

MOVE12 aimed for sustainable changes at both individual and environmental levels, At the meso level, regular meetings with community leaders and county decision-makers were planned, who were also part of the MOVE12 planning group, were organized to strengthen local implementation. At the macro level, public health and educational policymakers were invited to review program data at a project conference six months after the intervention. These efforts aimed to secure ongoing support and funding by demonstrating the program's wide-ranging benefits, including improvements in students' physical and mental health, development of social skills, a better school climate, enhanced concentration (facilitating faster learning), and increased engagement in theoretical lessons.

In order to increase students’ knowledge and awareness of the importance of physical activity for a healthy life, introductory information meetings (44) will be held at the respective schools. These meetings, led by the project leader and Principal Investigator (PI), will focus on the purpose and potential benefits of the MOVE-breaks. Given the uniqueness of the MOVE-breaks (short student-led physical activity sessions as a break in ordinary teaching sessions), a sub-goal is to help students who may initially be negative about physical activity change their predisposition through positive participation experiences (45). To foster students' self-efficacy in organizing and implementing MOVE-break sessions, PE teachers will provide a comprehensive 2 × 90-min introductory course. This course will include practical training and ongoing feedback for students before the intervention begins (46, 47). Aligned with self-determination theory (SDT), students will be able to set their own goals for the MOVE-break they lead. This autonomy is expected to enhance their motivation for participation (48). Structured practice under the supervision of the PE teacher will help build students' competence in planning, organizing, and evaluating MOVE-breaks. This competence-building is reinforced through social comparisons with peer pairs who share similar responsibilities during the pilot study, fostering confidence in their physical activity abilities (46). Finally, the implementation of student pairs, along with support from PE and teacher ambassadors, is designed to provide mutual support, addressing the need for relatedness. These structures and processes aim to maintain motivation through social encouragement and accountability (49).

At the environmental level, school managers will be encouraged to focus on supporting and motivating both teachers and students in the implementation of the MOVE-break, with the goal of eventually integrating it into the school's routine. This effort can be facilitated by featuring it as an agenda item in upcoming leadership and staff meetings. Additionally, we will encourage physical education teachers and other relevant teachers to support students and ensure the regular implementation of MOVE-breaks 1–2 daily. Tailored information and promotion will be used to foster a positive school culture around MOVE-breaks, relying on social support from leadership down to the student level. Additionally, the project group will encourage school leaders to actively promote physical activity in the daily school routine, both internally and in external forums, drawing inspiration from organizational development theory, which involves ongoing, iterative adjustments and rebalancing within organizational routines and processes (50).

To further assist schools in fostering a positive culture for implementing regular MOVE-breaks throughout the school day, participating schools will receive financial incentives to allocate to dedicated teacher ambassadors (Table 2). In terms of the physical environment (51), a compendium of physical activities and exercises (52) have been developed for seamless implementation in classroom settings with minimal furniture modifications, such as rearranging desks and chairs. To support this process, the project team created a digital activity guide featuring strength- and endurance-based exercises tailored for classroom use. Students designed their own MOVE-break session plans, incorporating images and detailed descriptions, and utilized classroom projectors to play follow-along dance videos (e.g., “Just Dance”). Additionally, each intervention class was provided with simple, portable equipment, such as beanbags, cards, and dice, enabling a variety of basic games, team exercises, and relay activities that are easy to organize and integrate into lessons.

#### 2.3.1 Intervention feasibility

To address scheduling challenges and classroom disruptions in MOVE12, the implementation of MOVE-breaks was designed with flexibility to accommodate the unique traditions, practices, and challenges of individual schools and classes. This collaborative approach, grounded in research-based strategies, promotes effective integration into diverse educational settings. The study design incorporates peer-led sessions (Table 2), where student pairs co-lead MOVE-breaks to foster autonomy, reduce teacher workload, and enhance peer accountability while maintaining classroom order (33, 53). Integration into the curriculum, such as fixed 10-min breaks during natural transitions in 90-min lectures, minimizes disruptions and optimizes participation (49). Classroom management protocols, including clear guidelines, designated movement areas, and student role assignments, maintain structure and discipline during activities (50). Regular feedback mechanisms will gather input from teachers and students on scheduling preferences and activity formats, enabling iterative refinements to improve feasibility and satisfaction (51). Lastly, providing simple, minimal equipment, such as portable items like beanbags or dice, ensures activities are quickly initiated and cleared, minimizing logistical hurdles and interruptions. These strategies collectively enhance the adaptability, effectiveness, and sustainability of MOVE12 across varied school contexts.

#### 2.3.2 Stakeholder engagement

To ensure the sustainability of MOVE12, stakeholder engagement (Table 2) will be a critical factor. Following the conclusion of MOVE12, a subsequent main intervention is planned, which will maintain the involvement of the same teacher ambassadors. Together with school leaders and additional teachers recruited for the main intervention, these stakeholders will receive close follow-up through regular meetings with the project leadership, both prior to and during the intervention period. After the main intervention, the project leader will establish ongoing collaboration by organizing periodic meetings with school leaders and teacher ambassadors, facilitated by the county municipality. These meetings aim to sustain engagement, share experiences, and develop strategies for integrating the intervention into long-term school routines.

### 2.4 Program production

Building on the methodologies and applications selected in Step 3, the fourth step of the IM protocol process entails the development of the intervention program, complemented by a detailed inventory of materials necessary for execution. The program production is divided into two primary phases:

#### 2.4.1 Preparation phase

This initial phase is dedicated to applying the findings from the previous theoretically based steps by developing a strategy to promote the initiative among students and teachers, and by preparing presentations and relevant tools/materials for the planned intervention. Distinct 60-min information sessions will be conducted for school leaders, teachers, and students to ensure each participant is thoroughly briefed on their roles and responsibilities. For these sessions, a PowerPoint presentation will be prepared, detailing the program's rationale, objectives, content, outcomes, and ethical considerations. Moreover, students will receive details on the MOVE-breaks, explaining its incorporation into a specific competency goal in physical education that requires planning, executing, and evaluating a personal training period. Additionally, a 5-min demonstration video showcasing various physical measurement techniques will be created for students. For teachers, we will create a promotional video lasting 5 min that emphasizes the benefits of incorporating brief MOVE-breaks into classroom settings (54).

#### 2.4.2 Practice phase

The next phase focuses on giving students practical-methodological practice in various ways to conduct MOVE-break sessions. It includes two 90-min sessions where students, in pairs, use the exercise compendium as a reference to plan, conduct, and assess their own 6–7-min MOVE-breaks for their peers, under the guidance of the physical education teacher. These practice sessions can be held in classrooms or suitable indoor/outdoor spaces nearby.

### 2.5 Program implementation plan

**Figure 3:**
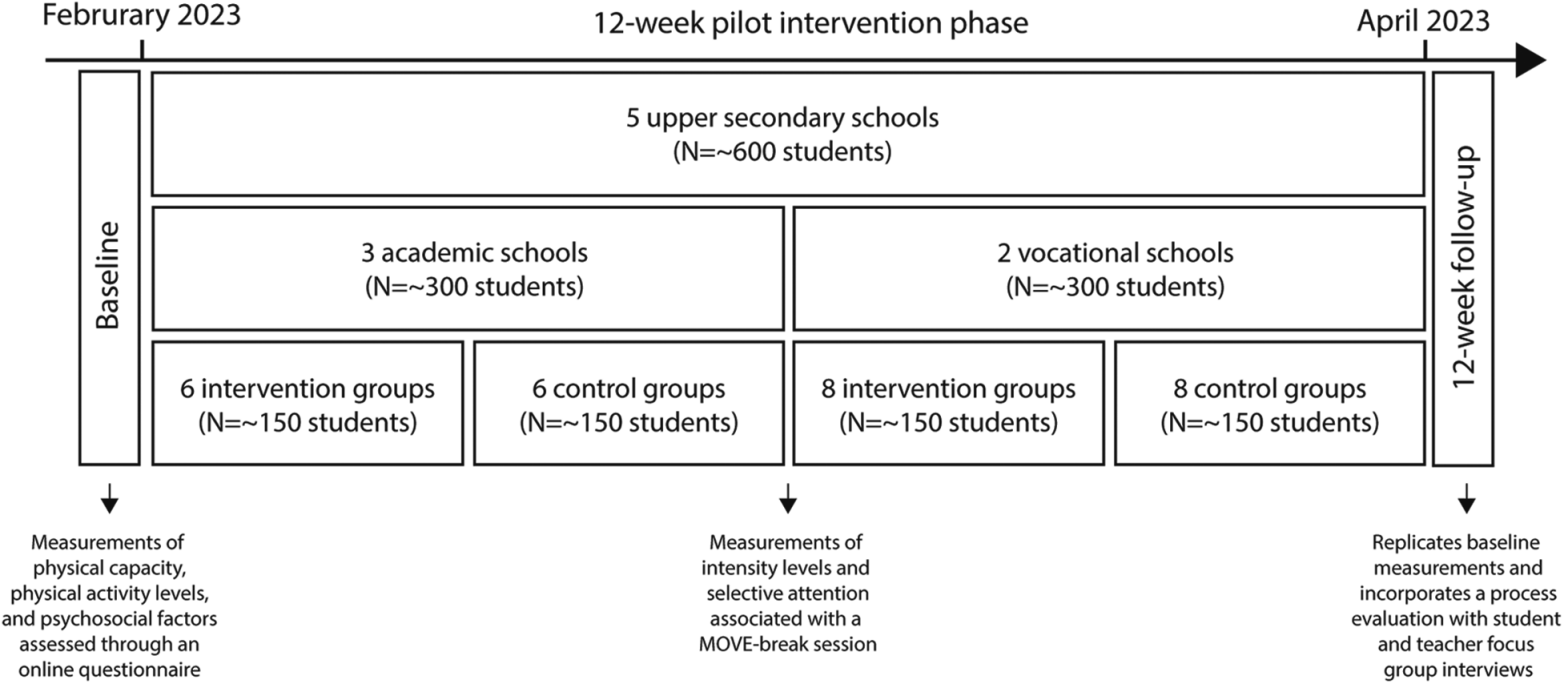
Timeline for the MOVE12 pilot intervention study.

The fifth step of the IM protocol process focused on planning the adoption, implementation, and sustainability of the intervention. This stage emphasizes engaging stakeholders to address barriers and refine strategies to maximize the intervention's reach and impact. Stakeholder discussions played a pivotal role in shaping the implementation plan. For example, feedback from school leaders and teacher ambassadors highlighted the need for financial incentives to encourage teacher ambassadors' active involvement. This input led to the allocation of dedicated funding to support their roles, ensuring consistent oversight and motivation during the intervention. Additionally, the needs assessment highlighted the importance of tailored communication strategies for parents to foster engagement and extend the program's impact beyond the classroom. In response, an information letter was developed to emphasize the benefits of MOVE-breaks in enhancing students' physical activity levels and overall well-being. As illustrated in Figure 3, the implementation of MOVE-breaks commenced following the completion of baseline measurements at the end of January 2023 and continued for a 12-week period, concluding in April 2023. This timeline allowed for a structured rollout while maintaining flexibility to address any unforeseen challenges. By incorporating the iterative refinements, the implementation plan was aligned with the practical needs of schools and stakeholders, enhancing the likelihood of sustained adoption and success.

#### 2.5.1 Study design

This protocol paper, developed using the intervention mapping (IM) framework, describes the planning of a 12-week cluster-randomized mixed-methods pilot study, incorporating both quantitative and qualitative data collection and analysis. The target population included first-year upper secondary school students from a broad region in southeastern Norway (three counties), aiming for balanced representation of academic and vocational programs. Invitations were sent to 27 upper secondary schools, and five agreed to participate (three academic, two vocational). One vocational school, twice the size of the others, contributed approximately 200 students, while each of the remaining four contributed about 100 students. Of 739 eligible students, 519 provided consent. The inclusion criteria for participation were students aged 16–17 years, with exclusion criteria defined as disabilities preventing participation and/or illnesses posing health risks.

#### 2.5.2 Randomization procedure

The MOVE12-pilot study was designed as a two-arm, three-level cluster randomized controlled trial (RCT), featuring an intervention group (IG) and a control group (CG), with participants organized within classrooms and schools. Participants were randomly assigned to either the IG or the CG (1:1) by the principal investigator (PI). At the three academic schools (class size: 25–30), four relatively homogeneous classes were identified, and randomization involved drawing from two boxes (one for group assignment, one for classes) in alternating turns until all classes were assigned. At the two vocational schools (class size: 12–17), classes were stratified by gender, size, and subject area before following the same procedure using four boxes (A–D). Twice as many groups were drawn from the vocational school that was double the size of the others.

#### 2.5.3 Intervention content

During the intervention period, the intervention groups were encouraged to incorporate at least two 6–7-min MOVE-breaks into their daily classroom sessions. The PE teacher, in collaboration with the participating teachers, coordinated the rotation of student pairs responsible for leading these sessions in various lectures. Additionally, teacher ambassadors played a crucial role in assisting with the implementation of the intervention. They worked closely with the Principal Investigator (PI) to provide support and encouragement to both the teachers and participating students. To support this process, the project team developed a digital activity guide featuring strength- and endurance-based exercises for the classroom. Students created their own MOVE-break session plans, adding images and descriptions. They also used computers to play follow-along dance videos (e.g., “Just Dance”) via the classroom projector. In addition, each class received simple equipment (beanbags, cards, dice) for basic games, team exercises, and relay activities.

### 2.6 Evaluation plan

The aim of MOVE12 is to increase students' physical activity levels in school through participation in 1–2 daily MOVE-breaks lasting 6–7 min each. This goal is pursued by enhancing their knowledge, fostering positive attitudes, boosting self-esteem, and nurturing intrinsic motivation at the individual level, while also focusing on providing opportunities and social support from teachers and peers (see Table 1). The findings from our planned evaluation will be reported in accordance with the CONSORT 2010 guidelines for randomized trials (55).

#### 2.6.1 Primary outcome measures

The primary outcome measures align with the study's core objective: promoting student participation in 1–2 daily MOVE-breaks (6–7 min each) during regular class sessions (Table 1). Feasibility and implementation will be assessed by evaluating students' adherence to MOVE-breaks and gathering insights into the subjective experiences of both students and teachers. These evaluations will be guided by changeable determinants such as knowledge, attitude, self-efficacy, basic psychological needs (autonomy, competence, relatedness), social support, school culture, and the physical environment. Data will be collected through focus group discussions at the 12-week follow-up, providing a comprehensive understanding of the intervention's effectiveness.

#### 2.6.2 Secondary outcome measures

Additionally, the study aims to evaluate potential effects of MOVE-breaks on physical fitness, psychosocial health parameters, and school-related factors through the following outcome measures:

- Attention and Concentration: Measured with the Eriksen Flanker Test (56) and the Stroop Test (57) before and after a designated MOVE-break session at the midpoint of the intervention.
- Heart Rate Measurements: Measured during a designated MOVE-break session at the midpoint of the intervention using the Polar Team Pro System (58).
- Physical Activity Level: Measured using the ActiGraph wGT3X-BT (59) at baseline and 12-week follow-up.
- Aerobic Fitness: Measured with the YMCA 3-min step test (60) at baseline and 12-week follow-up.
- Muscle Strength: Measured by (i) standing long jump (61) and ii) handgrip (dynamometer) (62) at baseline and 12-week follow-up.
- Postural Balance: Measured by two-leg standing, eyes closed (30 s) and one-leg standing, eyes open (30 s) (63) at baseline and 12-week follow-up.
- Flexibility: Measured by the sit-and-reach test (64) at baseline and 12-week follow-up.
- Sleep Quality: Measured by an online questionnaire (Nettskjema.no) at baseline and 12-week follow-up using four single items from a modified version of the Karolinska Sleep Questionnaire (65).
- Wellbeing: Measured by an online questionnaire (Nettskjema.no) at baseline and 12-week follow-up using the Warwick-Edinburgh Mental Wellbeing 7-item scale (66).
- Self-Efficacy: Measured by an online questionnaire (Nettskjema.no) at baseline and 12-week follow-up using a factor developed by Sørlie and Nordahl (67), based on Bandura (68).
- Learning Environment and Social Wellbeing in Class: Measured by an online questionnaire (Nettskjema.no) at baseline and 12-week follow-up using 13 items developed by Moos and Trickett (69), translated and processed by Sørlie and Nordahl (67).
- Social Isolation: Measured by an online questionnaire (Nettskjema.no) at baseline and 12-week follow-up using 21 items from the Social Skills Rating System by Gresham and Elliott (70), translated and processed by Sørlie and Nordahl (67).

### 2.7 Quantitative and qualitative data analysis

Data analysis will combine quantitative and qualitative approaches. To examine between-group differences in continuous outcome measures from baseline to the 12-week follow-up, a linear mixed model (LMM) will be applied. LMM addresses the nested data structure of MOVE12 (students within classrooms, classrooms within schools) by incorporating random intercepts to account for intra-class correlation and hierarchical clustering effects (71). Additionally, LMMs handle missing data robustly under the assumption of missing at random (72), a common scenario in school-based interventions. Within-group changes will be assessed using dependent *t*-tests, and potential associations between ordinal outcome measures will be explored through multiple and binary regression analyses. All statistical analyses will be conducted using STATA version 18.0 (73).

Qualitative methods will be used to identify key themes from the planned focus group interviews, guided by determinants such as knowledge, attitude, self-efficacy, basic psychological needs (autonomy, competence, relatedness), social support, school culture, and the physical environment. This qualitative approach aims to deepen our understanding of how these factors influence participants' experiences and engagement with the intervention, thereby informing both practical implementation and theoretical implications. Qualitative data will be analyzed thematically using NVivo 14 software to support and manage the analytic process (74).

*a priori* sample size calculations with G-Power (75) suggested that a sample of 580 participants would be sufficient to detect a moderate effect size (*d* = 0.3), assuming a two-group design, 80% power, and an alpha of.05.

## 3 Discussion

Recent meta-analyses and systematic reviews provide growing evidence supporting physical activity breaks in schools, demonstrating benefits for students' physical activity levels (76–78), attention (79), academic performance (80, 81), health-related quality of life (82), and aerobic fitness (19, 83). These findings underscore the classroom as a promising setting for promoting physical activity with potential benefits for learning outcomes and student well-being. However, it is equally important to recognize that some reviews report modest or inconsistent effects, including weak trends or null findings, particularly regarding long-term impact, feasibility, and contextual variations in implementation success. These inconsistencies reveal critical knowledge gaps that necessitate further investigation into targeted, sustainable, and scalable approaches (11).

The MOVE12 study addresses these gaps through the evaluation of a systematically designed, classroom-based intervention developed using the robust, theory-driven Intervention Mapping (IM) protocol. By targeting both individual and environmental determinants, MOVE12 promotes short, student-led physical activity breaks (MOVE-breaks) integrated seamlessly into the school day. The intervention incorporates evidence-based components, peer-led sessions, autonomy-supportive structures, and minimal resource requirements, to enhance feasibility, adaptability, and sustainability across diverse school settings. Its multi-level approach considers individual, interpersonal, and organizational factors, ensuring comprehensive evaluation while addressing real-world challenges that affect intervention effectiveness.

Guided by the socio-ecological model (SEM) and self-determination theory (SDT), MOVE12 represents an innovative approach to integrating physical activity into upper secondary school routines. The IM protocol enabled a systematic and theory-informed process for designing, implementing, and evaluating this health promotion intervention. One of the key strengths of using IM lies in its step-by-step framework, which ensures each phase is grounded in theoretical and empirical evidence (30). This structure is particularly valuable for addressing complex health behaviors like physical activity, which are influenced by interdependent factors at multiple levels, from individual motivation to school culture and broader policies.

The MOVE12 study aims to build on previous efforts by embedding regular, short physical activity breaks throughout the school day. While prior interventions have shown varying levels of success, the strategy seeks to create an environment that actively supports sustained physical activity. By incorporating MOVE-breaks into daily school routines, the intervention has the potential to enhance adherence to physical activity guidelines (84, 85), achieving the recommended 60 min of moderate-to-vigorous physical activity per day (76, 86). Additionally, the flexible design allows schools to tailor the program to their specific contexts, effectively addressing variations in resources, constraints, and classroom structures across academic and vocational programs.

This study also enhances the theoretical understanding of intervention design by integrating SEM and SDT to create supportive environments that not only allow, but actively encourage regular physical activity among adolescents (35, 87). The intervention emphasizes autonomy and peer support, core SDT constructs that are shown to enhance motivation, engagement, and sustainability of behavior change. Research supports this dual approach, as interventions fostering autonomy, competence, and relatedness are more likely to be effective and maintained over time (38, 43).

Despite its strengths, the application of the IM protocol in MOVE12 poses several challenges. A significant issue is ensuring the fidelity across diverse school environments, particularly given structural differences between academic and vocational study programs in terms of classroom (88). Academic students typically work at desks in traditional classrooms, whereas vocational students experience more diverse working methods, such as alternating between standing and sitting, and varied classroom environments like workshops, specialized workspaces, and classrooms that differ according to the subject area. Variations in resources, commitment, and existing cultures among schools could affect the consistency and effectiveness of the intervention's implementation. This highlights the need for adaptable yet structured implementation strategies that can accommodate various school environments/contexts without compromising the intervention's integrity (89, 90). Another challenge is securing engagement from all stakeholders, particularly teachers who are not directly involved as teacher ambassadors (91, 92). Their broader involvement is crucial for the sustainable integration of physical activity interventions in schools, as they play a significant role in shaping school culture and student behaviors (93).

The MOVE12 study offers valuable insights for the design and implementation of health promotion interventions in educational settings. Future research should focus on the scalability of such interventions, exploring factors like school size, diverse demographics, and varying levels of existing infrastructure. From a policy perspective, MOVE12 has the potential to inform school-based strategies that address physical inactivity, contributing to improved public health outcomes. The evaluation of the MOVE12 pilot intervention will assess the effectiveness of the intervention mapping protocol in establishing a foundation for sustainable, scalable, and impactful physical activity interventions in schools.

## Data Availability

The raw data supporting the conclusions of this article will be made available by the authors, without undue reservation.
